# Case of a Retention of Urine Removed by Electricity

**Published:** 1786

**Authors:** Samuel Snowden

**Affiliations:** Physician at Stroud, in Gloucestershire


III. *Caſe of a Retention of Urine removed by Electricity. Communicated in a Letter to Dr.* Simmons, *by* Samuel Snowden, *M. D. Phyſician at Stroud, in Glouceſterſhire.*

I TAKE the liberty to ſend you the following caſe, to be inſerted, if you pleaſe, in the London Medical Journal. It relates to a retention of urine removed by electricity; which being but ſlightly mentioned by authors, may, perhaps, merit attention. The patient, whofe cafe I mean briefly to recite, is between forty and fifty years of age. He had been for fome time, previous to the retention of urine, in an ill ftate of health, and fubject at intervals to pains in differenr parts of the body, which he called gouty, together with marks of confiderable debility: to be fhort, he was in that ftate which is commonly termed a breaking up of the conftitution.

It may not be foreign to the fubject to mention, that in July laft the patient was on a fudden feized with a convulfive motion of the left arm, which produced a ftrong contraction of the fore fingers, attended with extreme pain. The fore arm continued inceffantly in motion for many days. Bleeding, mofch, opium, and bark, were the principal remedies made ufe of. During this illnefs, an eruption was obferved on the face and hands, and, indeed, difperfed on fome other parts of the body: but upon the complaint leaving the part, the eruption became univerfally thick, affording great relief to the patient's general health. This eruption refembled the nettle rafh.

About the middle of September I vifited this patient for a retention of urine; and it may be remarked, that, in the intermediate fpace from his laſt illneſs to the preſent, he had been particularly attentive to his manner of living. His pulſe I found in a natural ſtate; his tongue clean and moiſt, with little thirſt; the heat of his body was not increaſed, and his ſleep and appetite were unimpaired: but he was coſtive, and complained of wandering pains, which, at times, were very ſevere, and affected, for the moſt part, the left hip joint. In this manner the diſeaſe went on for the ſpace of thirteen days, without any material incident occurring worth remarking; and it may be ſufficient to obſerve, that the remedies uſually recommended by authors were adopted, but without ſucceſs. The water was daily drawn off by Mr. Hughes, an ingenious ſurgeon of this place; and it may be remarked, that the catheter received no impediment at the times of introduction.

In the beginning of the retention, the patient, unwilling to ſubmit to the uſe of the catheter, ſuffered the bladder to be ſo much diſtended, as to occaſion moſt excruciating pain, which, however, ceaſed, upon the bladder being emptied; nor, indeed, was there ever after the leaſt inconvenience found from the diſtention of the bladder, although the water was was drawn off but once in four and twenty hours. The water ufually evacuated amounted to three pints.

Things remaining in this ftate, and feeing little good to be effected by internal remedies, from an utter diflike our patient had formed of them, I propofed a trial of electricity; to this he readily acquiefced; and having a machine of his own, it was ufed in the following manner — In the morning, on the thirteenth day of the complaint, the patient was fimply electrified, and while on the ftool, he was enabled to pafs a few fpoonfuls of water; many attempts were made to accomplifh this, when electricity was not ufed, but fruitlefs were the attempts; on which account, he was induced to have frequent accefs to the fame experiment through the day. On the evening of the fame day, I directed, that a few fhocks fhould pafs through the region of the bladder; but, from this method, no water paffed; and fo trifling was the difcharge of water through this day, that I judged it expedient to have recourfe to the catheter. In the morning, on the following day, the patient, having no opinion himfelf of fhocks, was again fimply electrified, and this had a better effect than on the preceding day,

 by by procuring a larger difcharge of water: the different times of electrifying through this day fufficiently unloaded the bladder, fo as to render the catheter unneceffary; nor indeed has electricity or the catheter been made ufe of fince. It may be noticed, that from the ufe of electricity, an eruption, of the fame kind as in the former illnefs, fhewed itfelf all over the body, but particularly on the face and hands. The pain of the hip became intolerable, fo much fo, as to oblige the patient to keep out of bed the beft part of many nights. A few weeks are elapfed, and he has had no return of the retention of urine.

